# Laying it on thick: a study in secondary growth

**DOI:** 10.1093/jxb/erab455

**Published:** 2021-10-16

**Authors:** Emma K Turley, J Peter Etchells

**Affiliations:** 1 Department of Biosciences, Durham University, South Road, Durham DH1 3LE, UK; 2 The Sainsbury Laboratory, Norwich Research Park, Norwich NR4 7UH, UK; 3 University of Manchester, UK

**Keywords:** Arabidopsis, auxin, cambium, cytokinin, phloem, procambium, stem cells, signalling, transcription factors, xylem

## Abstract

The development of secondary vascular tissue enhances the transport capacity and mechanical strength of plant bodies, while contributing a huge proportion of the world’s biomass in the form of wood. Cell divisions in the cambium, which constitutes the vascular meristem, provide progenitors from which conductive xylem and phloem are derived. The cambium is a somewhat unusual stem cell population in two respects, making it an interesting subject for developmental research. Firstly, it arises post-germination, and thus represents a model for understanding stem cell initiation beyond embryogenesis. Secondly, xylem and phloem differentiate on opposing sides of cambial stem cells, making them bifacial in nature. Recent discoveries in *Arabidopsis thaliana* have provided insight into the molecular mechanisms that regulate the initiation, patterning, and maintenance of the cambium. In this review, the roles of intercellular signalling via mobile transcription factors, peptide–receptor modules, and phytohormones are described. Crosstalk between these regulatory pathways is becoming increasingly apparent, yet the underlying mechanisms are not fully understood. Future study of the interaction between multiple independently identified regulators, as well as the functions of their orthologues in trees, will deepen our understanding of radial growth in plants.

## Teaser

We review the literature describing the molecular mechanisms by which the vascular cambium is initiated and maintained in Arabidopsis.

## Introduction

Plants exhibit an extraordinary capacity for growth and developmental plasticity, owing to their ability to maintain populations of continuously dividing stem cells in tissues called meristems. Two indeterminate meristems are established during embryogenesis. These are the root apical meristem (RAM), which gives rise to the subterranean root system, and the shoot apical meristem (SAM), from which all aerial tissues are derived. The elongation of plants along the apical–basal axis that results from RAM and SAM activity is termed primary growth. In addition, plants develop a variety of post-embryonic meristems, from which axillary branches, floral organs, and lateral roots are derived (Taiz and Zeiger, 2002). As the architectures of land plants (Embryophyta) became larger and more complex during the Devonian period ~400 million years ago, the development of specialized tissues for mechanical support and efficient long-distance transport of fluids became increasingly advantageous (Tonn and Greb, 2017). As a result, the ability to expand roots and stems along the radial axis (termed secondary growth) has evolved multiple times in the Embryophyte lineage, and can be observed in extant gymnosperms and the majority of dicotyledonous angiosperms (Spicer and Groover, 2010; Nieminen *et al.*, 2015).

**Fig. 1.**
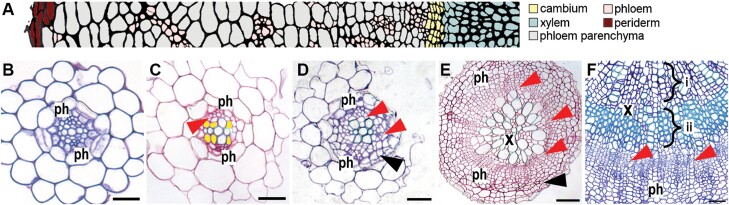
Secondary growth in Arabidopsis root and hypocotyl. (A) Cell types and their distribution in the Arabidopsis hypocotyl. (B–E) Transverse sections of Arabidopsis roots at different stages of secondary development. Red arrowheads mark cambial cell divisions. (B) Primary diarch pattern, in 7-day-old root, characterized by a central row of xylem with two adjacent phloem poles. (C) Formation of xylem vessels adjacent to the central xylem axis and initial cambial cell divisions (14-day-old root). Differentiating secondary xylem (marked yellow) acts as a cambium organizer, promoting adjacent cells to divide, thus initiating secondary growth. (D) In 18-day-old root, cambial divisions surround the xylem. Divisions in the cork cambium are apparent (black arrows). Secondary growth pushes the vascular cylinder through the cortex and epidermis. The radial pattern is complete in a 30-day-old root (E). (F) Transverse section through a 36-day-old hypocotyl showing xylem formed during the proportional growth phase (i) and xylem expansion growth phase (ii). Scales are 20 µm in (B–D); 50 µm in (E) and (F). X, xylem; ph, phloem.

Secondary growth arises from tightly controlled cell divisions in post-embryonic meristems known as the vascular and cork cambia. The vascular cambium, which is the focus of this review, gives rise to a network of interconnected transport cells and their supporting tissues, which span the entire primary plant body and its lateral organs. Uniquely among plant meristems, the vascular cambium harbours a single, bifacial stem cell in each radial file, which divides periclinally (parallel to the surface of the organ) to drive the development of distinct specialized tissues on opposing sides (Bossinger and Spokevicius, 2018; Shi *et al.*, 2019; Smetana *et al.*, 2019). Stem cells in the vascular cambium give rise to xylem centripetally and phloem centrifugally. Together, these dividing cells and their undifferentiated daughters (xylem and phloem progenitors) form a ‘cambial zone’, which is especially visible in transverse sections of mature Arabidopsis hypocotyls (Fig. 1A).

Xylem tissue is composed of conductive vessel elements that become hollow and vertically connected following programmed cell death (reviewed in Bollhöner *et al.*, 2012). The resulting channel facilitates the transport of a continuous acropetal stream of water and dissolved minerals. Interspersed with vessel elements are dead xylem fibres and living parenchyma cells, the latter of which store starches, oils, and tanniniferous compounds (Esau, 1977). Vessel elements and fibres possess thick secondary cell walls of cellulose and hemicellulose, reinforced with lignin biopolymers to confer a high tensile strength. This allows xylem to mechanically support plant tissues as they elongate and withstand the negative pressures required for water transport (Taiz and Zeiger, 2002). On the opposing side of the vascular cambium, the phloem supports the bidirectional transport of sugars, proteins, amino acids, and other metabolites. Like xylem, phloem is composed of multiple specialized cell types, including conductive sieve elements (Fig. 1A). As phloem progenitors exit the cambial zone, organelles including the nucleus, vacuole, and cytoskeleton are degraded, and perforated sieve plates form to establish continuous connections between vertically adjoining cells (Furuta *et al.*, 2014). Sieve elements have highly restricted metabolic activities and are thus supported by companion cells, to which they are connected via plasmodesmata. Other supporting cells include the phloem parenchyma and fibres, which fulfil storage roles and provide mechanical support, respectively (Esau, 1977). In addition to their roles in transporting photoassimilates and other nutrients, the importance of phloem highways for carrying long-distance RNA, phytohormone, and electrical signals is becoming increasingly apparent (Hilleary and Gilroy, 2018; Johns *et al.*, 2021). This gives phloem tissue a central role in both plant growth and stress responses.

While the vascular cambium produces xylem and phloem, the outer cork cambium (also known as the phellogen) contributes to radial growth by producing protective tissue (Fig. 1A, D, E). In a layer known as the periderm, the cork cambium divides periclinally to produce phelloderm centripetally and phellem centrifugally. The dead cork cells that comprise the phellem have walls layered with suberin and lignin, making them difficult for insects and phytopathogens to penetrate (Wunderling *et al.*, 2018; Campilho *et al.*, 2020). Therefore, replacement of the epidermis with phellem in woody stems and roots protects the internal transport tissues from environmental stress by reducing water loss and susceptibility to biotic threats.

## *Arabidopsis thaliana* as a secondary growth model

While secondary growth is evidently important for plant development, the position of cambial meristems deep within internal tissues has hampered efforts to study their activity. Nevertheless, advances in plant genetics, tissue processing, and microscopy have recently accelerated vascular development research (reviewed in Nieminen *et al.*, 2015; Lehmann and Hardtke, 2016; Fischer *et al.*, 2019; Wang, 2020). Despite its herbaceous nature (i.e. lack of a persistent woody stem), Arabidopsis exhibits secondary thickening of its roots, hypocotyls, and inflorescence stems. Comparative anatomical studies have revealed striking similarity between the concentric patterns of xylem–cambium–phloem in the hypocotyls of Arabidopsis, stems of angiosperm trees, and storage roots of numerous crop species (Chaffey *et al.*, 2002; Campilho *et al.*, 2020; Hoang *et al.*, 2020*b*). However, notable differences include the inability of Arabidopsis to form annual growth rings and its lack of ‘ray’ systems, which facilitate the radial transport of resins and gums in mature trees (Esau, 1977; Chaffey *et al.*, 2002). Even so, key genetic regulators of vascular development are conserved between Arabidopsis and its distant tree and root crop relatives (Wunderling *et al.*, 2018; Hoang *et al*., 2020*a*, *b*), as discussed later in this review.

## A robust primary pattern sets the stage

Before secondary growth commences in any plant, a precise primary vascular pattern is established. In Arabidopsis, the initiation of vascular stem cells and the specification of xylem and phloem in the embryo have been well characterized (reviewed in Miyashima *et al.*, 2013; De Rybel *et al.*, 2016). At the late globular stage of embryogenesis, vascular stem cells and surrounding pericycle tissue arise from the periclinal divisions of four preprocambial initials. Further division and differentiation of these cells during the subsequent heart stage give rise to a stereotypical radial pattern of cell types, which is mimicked in the post-embryonic root. This arrangement includes five or six xylem vessels in a single file, spanning the diameter of the stele (Baum *et al.*, 2002). The central metaxylem has highly lignified, pitted cell walls, while their smaller protoxylem neighbours have spiral wall thickenings. Either side of these central xylem cells is a pool of procambium cells and a phloem pole containing sieve element precursors (Rodriguez-Villalon *et al.*, 2014). Having two strands of protoxylem, this primary pattern is referred to as a diarch (Fig. 1B).

**Fig. 2.**
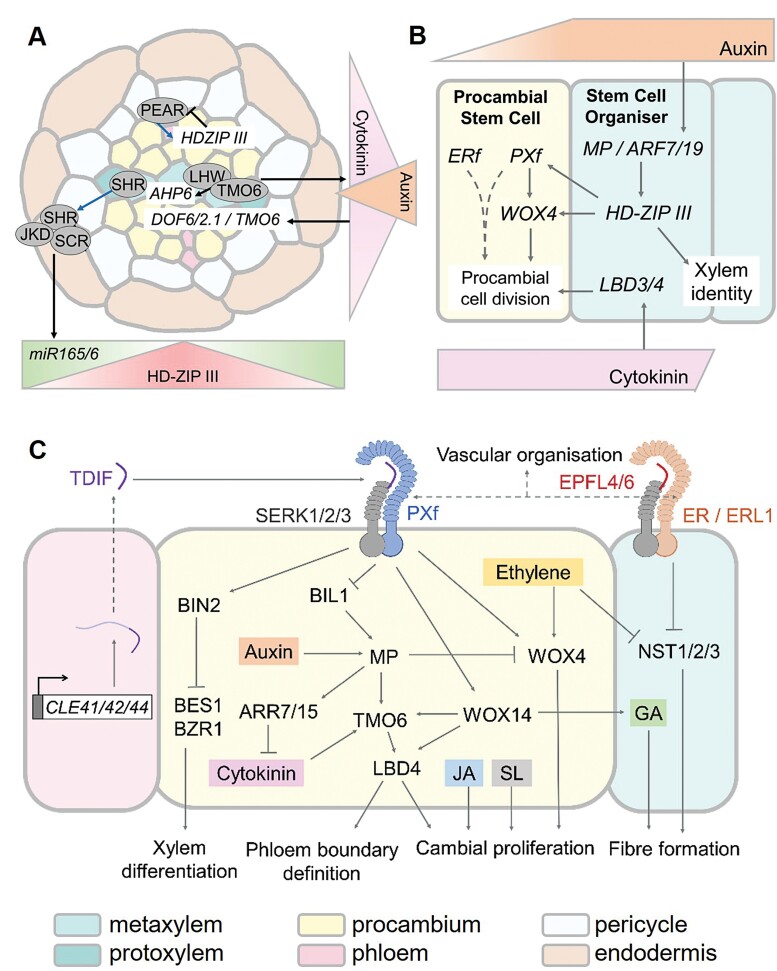
Mechanisms of secondary growth regulation in Arabidopsis. (A) Establishment of diarch vascular pattern in the primary root. (B) Initiation of secondary growth in roots, during which a stem cell organizer of xylem identity promotes division of neighbouring procambial cells. (C) Maintenance of the vascular cambium and organized development of secondary vasculature, involving integration of ligand–receptor pairs and phytohormone signalling. Phytohormones are represented by coloured boxes: JA, jasmonic acid; SL, strigolactone; GA, gibberellic acid. Triangles represent concentration gradients of phytohormones or gene products (A, B). Blue arrows represent protein movement in (A); black arrows show positive interactions; blunt lines show inhibition. Dashed lines represent unknown mechanism of interaction (B, C) or TDIF processing (C).

The formation of a diarch vascular cylinder is heavily dependent on intercellular signalling (Fig. 2A). Being one of the most important and extensively studied growth substances in plant development (Leyser, 2018), it is unsurprising that the phytohormone, auxin, has been implicated in this process. AUXIN RESPONSE FACTOR 5 (ARF5), also known as MONOPTEROS (MP), is released from its Aux/indole-3-acetic acid (IAA) inhibitor, BODENLOS (BDL), when cotyledon-derived auxin arrives at the vascular initials during embryogenesis (Weijers *et al.*, 2006). MP is essential for early procambium formation, given that *mp* loss-of-function mutants were deficient in embryonic provascular cell divisions (Hardtke and Berleth, 1998). In addition, expression of a mutated, stabilized version of BDL conferred a rootless phenotype (Weijers *et al.*, 2006), highlighting the importance of auxin signalling and MP function for root vascular development.

The cell to cell movement of transcription factors and miRNAs is known to play crucial roles in the establishment of root vascular patterns (De Rybel *et al.*, 2016). The GRAS transcription factor SHORT ROOT (SHR) is expressed in the stele of developing roots and moves outwards to the endodermis, where it induces the expression of another GRAS protein, SCARECROW (SCR) (Nakajima *et al.*, 2001). By binding SCR and the zinc finger BIRD transcription factors, JACKDAW (JKD) and BALDIBIS (BIB), SHR is sequestered in the nucleus of endodermal cells (Koizumi *et al.*, 2012; Long *et al*., 2015, 2017). Here, SHR binds the promoters of *MIR165* and *MIR166*, thereby inducing the expression of cell to cell diffusible miRNA, miR165/6 (Carlsbecker *et al.*, 2010). The five vascular-expressed class III HOMEODOMAIN LEUCINE ZIPPER (HD-ZIP III) transcription factors, REVOLUTA (REV), PHABULOSA (PHB), PHAVOLUTA (PHV), ARABIDOPSIS THALIANA HOMEOBOX 8 (ATHB8), and CORONA (CNA/ATHB15), are known targets of miRNA165/6-mediated post-transcriptional silencing (Mallory *et al.*, 2004; Smetana *et al.*, 2019). Accordingly, HD-ZIP III protein concentrations across the stele exhibit a gradient opposing that of their miRNA inhibitors, with abundance peaking in the stele centre and decreasing towards the pericycle (Carlsbecker *et al.*, 2010; Fig. 2A). *phb-1d* gain-of-function roots demonstrated ectopic development of metaxylem in place of protoxylem, while simultaneous knockout of four HD-ZIP III members triggered ectopic protoxylem marker gene expression in the centre of the xylem axis (Carlsbecker *et al.*, 2010). Thus, in a wild-type root, it is predicted that high HD-ZIP III levels promote metaxylem differentiation, whereas low levels promote protoxylem differentiation. This mechanism of HD-ZIP III-mediated xylem patterning is predicted to be similarly reflected in the Arabidopsis embryo (De Rybel *et al.*, 2016).

Beyond HD-ZIP IIIs, heterodimers of basic helix–loop–helix (bHLH) transcription factors were found to be necessary for the establishment of primary vasculature in Arabidopsis embryos (Schlereth *et al.*, 2010). These heterodimers comprise LONESOME HIGHWAY (LHW), or LHW-LIKE 1 (LHL1) proteins, and MP-induced TARGET OF MONOPTEROS 5 (TMO5), or TMO5-LIKE (T5L) proteins (Schlereth *et al.*, 2010; De Rybel *et al.*, 2013). In a screen for mutants with compromised cell fate specification, *lhw*-deficient primary roots had a reduced number of vascular cells and displayed an abnormal monarch pattern with only one protoxylem strand (Ohashi-Ito and Bergmann, 2007). A similar phenotype was later observed in *tmo5 t5l1* roots, and the formation of heterodimers between proteins of the LHW and TMO5 clades was confirmed in xylem precursor cells (De Rybel *et al.*, 2013). In the embryo and primary root, the LWH–TMO5 module stabilizes growth and patterning of vascular tissue via regulation of cytokinin signalling. LHW–TMO5 up-regulates expression of cytokinin biosynthesis genes, *LONELY GUY3* (*LOG3*) and *LOG4*, generating a cytokinin gradient that peaks in the xylem-adjacent procambium (De Rybel *et al.*, 2014). Here, cytokinin promotes expression of the transcription factor gene, *DNA-BINDING ONE FINGER2.1* (*DOF2.1*), which drives procambial cell division alongside its two homologues, *TMO6* and *DOF6* (Smet *et al.*, 2019). At the same time, *ARABIDOPSIS HISTIDINE PHOSPHOTRANSFER PROTEIN 6* (*AHP6*), a repressor of cytokinin signalling, is cell autonomously induced by LHW–TMO5 which act downstream of auxin signalling to maintain the primary xylem in a non-dividing state (Bishopp *et al.*, 2011; Ohashi-Ito *et al.*, 2014).

Recently, TMO6 and DOF6 were assigned to a group of cytokinin-inducible PHLOEM EARLY DOF (PEAR) proteins, which promote the division of protophloem sieve elements in young roots (Miyashima *et al.*, 2019). PEARs move intercellularly and up-regulate the expression of HD-ZIP IIIs in neighbouring cells. In turn, these inhibit PEAR expression to form a negative feedback loop, inhibiting periclinal division in the phloem-adjacent internal region of the root (Miyashima *et al.*, 2019). Overall, the interlinking of auxin and cytokinin signalling via HD-ZIP IIIs, LHW–TMO5, and DOF proteins facilitates the establishment of a robust primary vascular pattern. This is essential for defining domain boundaries for subsequent cell division.

## Procambial cells divide at the onset of radial growth

Soon after the establishment of diarch vascular bundles, radial growth initiates in the procambium, and secondary xylem is formed opposite the phloem poles (Esau, 1977; Baum *et al.*, 2002). Pioneering cell lineage tracing experiments in the Arabidopsis root have pin-pointed secondary growth initiation to the divisions of xylem-adjacent procambial cells that occur 5–6 d after germination (Smetana *et al.*, 2019) and ~15–18mm from the root tip (Ye *et al.*, 2021), although the exact timing of this transition is dependent on growth conditions (see, for example, Thamm *et al.*, 2019; Fig. 1C, D). HD-ZIP III transcription factors are known to promote this early vascular proliferation. Expression of these genes, together with auxin signalling via MP, ARF7, and ARF19, defines a ‘stem cell organizer’ (yellow cells in Figs 1C and 2B) that is continually renewed as the procambium divides. In support of this model, suppression of HD-ZIP III function by chemical induction of *MIR165*, thus targeting *HD-ZIP III* transcripts for degradation, resulted in scattered cambial divisions, erratic xylem formation, and mitotic re-entry of previously quiescent xylem (Smetana *et al.*, 2019). Following the initiation of procambial divisions, phloem-adjacent pericycle cells divide, pushing out the phloem region to generate an oblong-shaped stele (Fig. 1C; Baum *et al.*, 2002). Divisions in the pericycle adjacent to the xylem poles subsequently contribute cells to generate a continuous cylinder of vascular cambium, a process that is similarly observed in young hypocotyls (Fig. 1D, E; Lehmann and Hardtke, 2016).

**Fig. 3.**
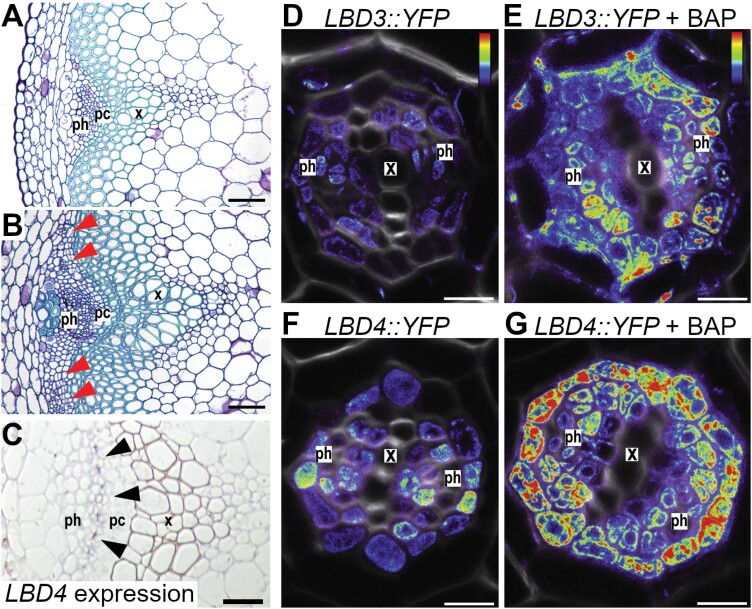
Secondary growth in the stem, and contrasting *LBD3/4* expression patterns in stems and roots. (A, B) Transverse sections of fascicles in Arabidopsis stems at 36 d. (A) Section from 2cm above the rosette showing a discrete vascular bundle with no secondary growth. (B) Section adjacent to the rosette showing secondary initiation, including cambial cell divisions (red arrowheads) adjacent to the fascicle. (C) *In situ* hybridization with the antisense *LBD4* probe marking the phloem–procambium boundary (Smit *et al.*, 2020). (D–G) *LBD3:YFP* and *LBD4:YFP* throughout the vascular cylinder in both the presence (E, G) and absence (D, F) of a 24h cytokinin treatment. Expression is higher in cytokinin-treated roots. Reprinted from Current Biology 31, Ye L, Wang X, Lyu M, Siligato R, Eswaran G, Vainio L, Blomster T, Zhang J, Mähönen AP. Cytokinins initiate secondary growth in the Arabidopsis root through a set of LBD genes. 3365–3373, Copyright (2021), with permission from Elsevier. Scales are 50 µm (A, B), 30 µm (C) and 10 µm (D–G). ph, phloem; pc, procambium; and x, xylem.

In contrast to the diarch patterns of immature roots and hypocotyls, primary vascular tissues in the Arabidopsis inflorescence stem are arranged in discrete vascular bundles (or fascicles). In a wild-type stem, 5–8 fascicles are derived from procambial cells below the SAM and arranged around a central pith (Fig. 3A), as is typical in dicots and gymnosperms (Fischer *et al.*, 2019). Secondary growth initiates in the procambium of vascular bundles, forming fascicular cambium. Meristematic activity also extends to the interfascicular regions, where stem cells are formed *de novo* from differentiated parenchyma cells or those in the inner endodermal layer (known as the starch sheath) (Agusti *et al.*, 2011*b*; Fig. 3B). The precise location of interfascicular cambium initiation is dependent on the stem’s developmental stage, with a gradual shift towards the cortex being observed as the stem apex is approached (Sehr *et al.*, 2010). Clusters of fascicular cambia are subsequently connected to form a continuous cambial ring in mature stems, from which secondary xylem and phloem are derived.

## Intercellular signalling via TDIF–PXY organizes secondary growth

Following the initiation of secondary growth, cell to cell communication remains integral for maintaining the activity of the vascular cambium. Coordinated regulation of cambial cell division and differentiation requires integration of multiple external signals, including those from the extracellular matrix and adjacent cell layers (Busch *et al.*, 2010; De Rybel *et al.*, 2016; Trewavas, 2021). The molecular mechanisms governing cambial maintenance are topics of ongoing research (for reviews, see Fischer *et al.*, 2019; Bagdassarian *et al.*, 2020; Wang, 2020).

Peptide perception by transmembrane receptor(-like) kinases (RK/RLK) is a key signalling mechanism underpinning vascular cambium activity. The ligand–receptor pair comprising TRACHEARY ELEMENT DIFFERENTIATION INHIBITORY FACTOR (TDIF) and PHLOEM INTERCALATED WITH XYLEM (PXY), also known as TDIF RECEPTOR (TDR), is an extensively studied and multifunctional signalling module in this context (Fig. 2C). As part of the largest RLK family in Arabidopsis (Becraft, 2002), PXY possesses an extracellular ligand-binding domain with 21 leucine-rich repeats (LRRs), a single-helix transmembrane domain, and a cytoplasmic kinase domain that is activated upon TDIF perception (Fisher and Turner, 2007; Hirakawa *et al.*, 2008). *PXY* has two close homologues in Arabidopsis, *PXY-LIKE 1* (*PXL1*) and *PXL2* (Fisher and Turner, 2007; Etchells *et al.*, 2013), collectively referred to as the PXY family (PXf).

**Fig. 4.**
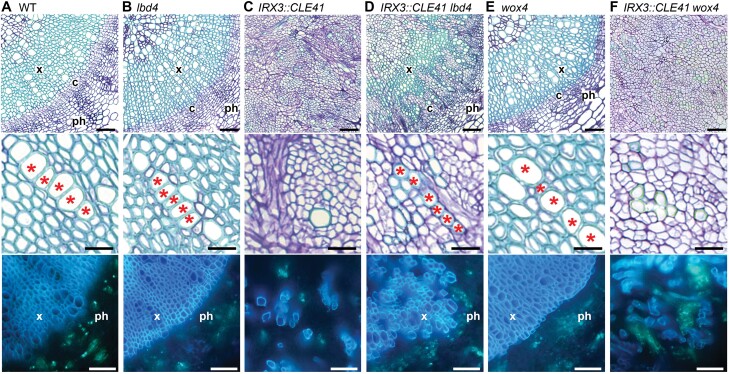
*wox4* and *lbd4* suppression of *IRX3::CLE41* phenotypes. (A) Wild type, (B) *lbd4*, (C) *IRX3::CLE41*, (D) *IRX3::CLE41 lbd4*, (E) *wox4*, and (F) *IRX3::CLE41 wox4* hypocotyls. Upper and middle panels show thin sections, stained with toluidine blue; the lower panel shows aniline blue hand sections. In aniline blue-stained sections, callose in the sieve plates fluoresces green, marking phloem. Lignin in secondary cell walls autofluoresces blue, marking the xylem. Red asterisks in (D) (*IRX3::CLE41 lbd4*) denote a file of differentiated xylem absent from *IRX3*::*CLE41* (C). Thus *lbd4* suppresses the organization defects in *IRX3::CLE41*. *IRX3::CLE41 wox4* lines (F) lack organization, but differentiated cell types are close together, suggesting a reduction in stem cells (lower panel). Thus *wox4* suppresses stem cell overproliferation observed in *IRX3::CLE41*. Scale bars are 50 μm in upper and lower panels; 20 μm in middle panels. X, xylem; ph, phloem; c, cambium.

*PXY* is expressed in vascular tissues throughout the plant body, including in leaf veins, inflorescence stems, hypocotyls, and root steles. This expression specifically localizes to the xylem side of the vascular cambium (Hirakawa *et al.*, 2008; Etchells and Turner, 2010*a*, *b*; Shi *et al.*, 2019; Smetana *et al.*, 2019; Wang *et al.*, 2019). In contrast, the genes encoding the PXY ligand, *CLAVATA3/ENDOSPERM SURROUNDING REGION 41* (*CLE41*), *CLE42*, and *CLE44* (Ito *et al.*, 2006), are expressed in the phloem (Hirakawa *et al.*, 2008; Etchells and Turner, 2010*a*). CLE41/42/44 peptides are 88–101 amino acids in length and are cleaved by a currently unknown mechanism to yield the TDIF dodecapeptide (Ito *et al.*, 2006). X-ray crystallography demonstrated that TDIF specifically bound PXY by interacting with the inner concave surface of the receptor’s LRR domain (Morita *et al.*, 2016; Zhang *et al.*, 2016*a*), following earlier identification of this interaction by photoaffinity labelling (Hirakawa *et al.*, 2008). Further structural and genetic studies revealed that, like other RLKs, PXY activation required SOMATIC EMBRYOGENESIS RECEPTOR KINASE (SERK) co-receptors, which associated with PXY in a ligand-dependent manner at the plasma membrane (Zhang *et al*., 2016*a*, *b*). An identifying feature of *pxy* loss-of-function mutants is their lack of a continuous cambial zone and resulting intercalation of xylem and phloem (Fisher and Turner, 2007; Wang *et al.*, 2019). Meanwhile, phloem-specific overproduction of TDIF in *SUC2::CLE41* stems resulted in enhanced, yet organized, vascular proliferation (Etchells and Turner, 2010*a*). In the same study, overexpression of *CLE41* via the *35S* or xylem-specific *IRREGULAR XYLEM 3* (*IRX3*) promoter triggered disorganized vascular development (Fig. 4A, C). Considering this, a phloem-derived TDIF signal is thought to convey positional information to PXY in order to maintain the activity and bifacial nature of cambial stem cells (Etchells and Turner, 2010*a*, *b*; Etchells *et al.*, 2016). Presently, this is understood to occur via three distinct pathways.

Firstly, PXY has an established role as a suppressor of cell differentiation. The TDIF peptide was originally identified as an inhibitor of xylem vessel differentiation when applied to *Zinnia* cell cultures (Ito *et al.*, 2006), a finding that was later replicated in Arabidopsis hypocotyls (Whitford *et al.*, 2008) and leaf disc cultures (Kondo *et al.*, 2015). Consistently, *35S:CLE41* plants (in which TDIF–PXY signalling is enhanced) showed reduced expression of the xylem development marker, *IRX3* (Etchells and Turner, 2010*a*). In addition, ectopic xylem differentiation and lignification of parenchyma cells were evident in *pxy* inflorescence stems (Etchells *et al.*, 2016). In wild-type plants, activation of glycogen synthase kinase 3 (GSK3) proteins actively suppresses xylem differentiation downstream of TDIF–PXY. Förster resonance energy transfer (FRET)-based analyses revealed PXY association with the GSK3, BRASSINOSTEROID INSENSITIVE 2 (BIN2), at the plasma membrane (Kondo *et al.*, 2014). In response to brassinosteroid perception, BIN2 is known to phosphorylate the transcription factors BRASSINAZOLE RESISTANT 1 (BZR1) and BRI1-EMS-SUPPRESSOR 1 (BES1), targeting them for proteolytic degradation (He *et al.*, 2002). These transcription factors redundantly promote xylem and phloem differentiation in cell culture systems (Saito *et al.*, 2018). PXY-mediated enhancement of BIN2 kinase activity is thus thought to promote phosphorylation and subsequent destabilization of BES1/BZR1, which is consistent with the reduced nuclear localization of BZR1–green fluorescent protein (GFP) observed in TDIF-treated seedlings (Kondo *et al.*, 2014). Through this BIN2–BZR1/BES1 pathway, PXY signalling protects cambial stem cells from differentiation, thereby maintaining a pool of dividing cells for secondary growth.

Secondly, TDIF–PXY promotes vascular proliferation by targeting the cambium-expressed *WUSCHEL-RELATED HOMEOBOX* (*WOX*) transcription factor genes, *WOX4* and *WOX14* (Ji *et al.*, 2010; Etchells *et al.*, 2013). Such CLE–RLK–WOX signalling modules have been repeatedly observed in plants and are known to regulate SAM and RAM maintenance (Schoof *et al.*, 2000; Stahl and Simon, 2009; Lee and Torii, 2012). Wild-type Arabidopsis seedlings incubated with TDIF exhibited an increase in procambial cell number and PXY-dependent up-regulation of *WOX4* and *WOX14* expression (Hirakawa *et al.*, 2010; Etchells *et al.*, 2013). Furthermore, *WOX4-*deficient plants had a reduced number of cells in their root and stem vascular bundles—a phenotype that was enhanced by simultaneous knockout of *WOX14* (Etchells *et al.*, 2013; Zhang *et al.*, 2019). Given that *wox4* and *wox4 wox14* mutants showed no defects in vascular organization (Fig. 4E, F), the TDIF–PXY–WOX4/14 module for regulation of cambial cell division is suggested to act in parallel to those regulating xylem differentiation (Etchells *et al.*, 2013).

Thirdly, a function for TDIF–PXY in defining the boundaries between vascular tissues was recently uncovered. Through loss- and gain-of-function approaches, *LATERAL ORGAN BOUNDARIES DOMAIN 4* (*LBD4*) and *WOX14* were identified as positive regulators of procambium activity in Arabidopsis roots (Zhang *et al.*, 2019). Later, these transcription factors were found to be part of a PXY-regulated feedforward loop that mediated cell proliferation and vascular bundle shape in inflorescence stems (Smit *et al.*, 2020). Strikingly, *lbd4* was found to be required for *IRX3:CLE41* phenotypes (Fig. 4C, D). Downstream of PXY, WOX14 functions with the auxin- and cytokinin-inducible DOF transcription factor, TMO6 (Schlereth *et al.*, 2010; Miyashima *et al.*, 2019) to promote the expression of *LBD4* along the phloem–procambium boundary (Smit *et al.*, 2020). Disruption of this pattern in *lbd4 IRX3::LBD4* stems (in which *LBD4* expression was confined to the xylem) resulted in a loss of the characteristic phloem arc and reduced expansion of fascicles along the radial axis (Smit *et al.*, 2020). Through this indirect regulation of *LBD4*, PXY is thought to regulate vascular organization by defining boundaries and amplifying cell divisions on the phloem side of the procambium.

## Cytokinin and the LBD family drive vascular development

Cytokinin is critical for cambium initiation. Indeed, simultaneous loss of four ATP/ADP ISOPENTENYLTRANSFERASE (IPT) enzymes, required for cytokinin biosynthesis (Miyawaki *et al.*, 2006), abolished secondary growth initiation in primary roots (Matsumoto-Kitano *et al.*, 2008). The vascular cambium was restored to *ipt* mutants by exogenous cytokinin application. This rescue was dependent on the presence of *LBD3* and *LBD4*, the expression of which is rapidly induced upon cytokinin treatment (Ye *et al.*, 2021; Fig. 3D–G). The expression of additional homologues, *LBD1* and *LBD11*, increased subsequently. Induction of the former pair occurred in the presence of protein synthesis inhibitors, demonstrating that these genes were primary cytokinin targets as their induction did not rely on the synthesis of intermediates. Thus, *LBD4* and its close relatives, *LBD3*, *LBD1*, and *LBD11*, act together to regulate radial growth by controlling cell division in the cambium. In support of this, loss of these four transcription factors resulted in considerable reductions in root secondary growth (Ye *et al.*, 2021).

Evidence suggests that a subset of LBD genes also influence cell size. Induction of *LBD1*, *LBD3*, or *LBD11* in young roots resulted in a marked increase in radial area of vascular cells, while their loss was characterized by a reduction (Ye *et al.*, 2021). One explanation for this combination of phenotypes is that cellular growth is closely interlinked with cell cycle progression (Sablowski and Carnier Dornelas, 2014). Therefore, these three *LBD* genes may drive division by first promoting cell enlargement. While cytokinin is primarily associated with cell division, increases in cell size have yet to be reported following its application during secondary growth. Also, evidence from the stem suggesting that *LBD4* expression marks the phloem–cambium boundary (Fig. 3C) in a PXY-mediated mechanism (Smit *et al.*, 2020) does not hold for cambium initiation in the root vascular cylinder, where *LBD3* and *LBD4* promoter activity is widespread (Fig. 3D–G). Perhaps the simplest explanation for these contradictions is that *LBD4* is multifunctional, with modified outputs depending on the developmental context.

## ERECTA receptors regulate vascular proliferation and fibre formation

Since the characterization of PXY, further RLKs with roles in vascular development have been identified (Agusti *et al.*, 2011*b*; Uchida and Tasaka, 2013; Wang *et al.*, 2013; Gursanscky *et al.*, 2016), although the components and interactions of their corresponding signalling pathways in vascular development are less well understood. Among these, ERECTA (ER) receptors are known to regulate cell division and xylem fibre formation during secondary growth (Ragni *et al.*, 2011; Uchida and Tasaka, 2013; Ikematsu *et al.*, 2017; Milhinhos *et al.*, 2019). *ER* encodes an RLK with 20 LRRs in its extracellular domain (Torii *et al.*, 1996), and possesses two close homologues, *ER-LIKE 1* (*ERL1*) and *ERL2* (Shpak *et al.*, 2004). This trio is collectively referred to as the ER family (ERf). *ER* was first cloned over two decades ago (Torii *et al.*, 1996), and has subsequently been implicated in a surprisingly diverse array of processes, including stomatal patterning (Shpak *et al.*, 2005), elongation of aerial organs (Woodward *et al.*, 2005; Chen *et al.*, 2013), reproductive development (Shpak *et al.*, 2004; Pillitteri *et al.*, 2007), meristem maintenance (Uchida *et al.*, 2012), leaf morphogenesis (Tameshige *et al.*, 2016), and responses to necrotrophic pathogens (Llorente *et al.*, 2005; Jordá *et al.*, 2016; Cai *et al.*, 2021). The ligands for ERf receptors reside within the family of EPIDERMAL PATTERNING FACTOR (EPF)/EPFL-LIKE (EPFL) peptides. These range from 45 to 75 amino acids in length and are encoded by 11 genes in Arabidopsis (Kondo *et al.*, 2010; Sugano *et al.*, 2010; Takata *et al.*, 2013). However, only a few receptor–ligand combinations have been studied in depth.

The influence of ERf signalling on plant development is seemingly dependent on the organ and *ER/ERL* genes in question. In inflorescence stems, the *ER* promoter is active in the xylem, phloem, and endodermis (Uchida *et al.*, 2012). *er*f triple mutants contained fewer cells within their stem vascular bundles, implicating ER/ERL receptors as positive regulators of vascular proliferation (Etchells *et al.*, 2013). Similarly, *er erl1* stems showed defects in procambium maintenance, a phenotype that was rescued by phloem-specific (but not xylem-specific) expression of *ER* (Uchida and Tasaka, 2013). The candidate ligands perceived by the ERf in this context are EPFL6 and EPFL4, also known as CHALLAH (CHAL) and CHAL-LIKE 2 (CLL2), respectively, which are highly expressed in inflorescence stems (Abrash *et al.*, 2011; Uchida *et al.*, 2012). The physical binding of ER to EPFL4/6 was confirmed by co-immunoprecipitation, while endodermis-specific expression of *EPFL4* or *EPFL6* rescued the *er*-like dwarf and compact inflorescence phenotypes of *epfl4 epfl6* double mutants (Uchida *et al.*, 2012). Thus, EPFL peptides secreted from the endodermis are hypothesized to signal to ERf receptors in the phloem to promote both inflorescence elongation and vascular development.

Conversely, a role for ERf receptors as suppressors of secondary growth has been uncovered in hypocotyls. *ER*, *ERL1*, and *ERL2* expression is observed in most hypocotyl cell types and peaks in the cambium, xylem initials, and periderm, although *ERL2* promoter activity is only evident in mature hypocotyls (Ikematsu *et al.*, 2017; Wang *et al.*, 2019). In contrast to the stem, *EPFL4/6* expression in hypocotyls is greatest in the xylem parenchyma and differentiating xylem, suggesting that most active EPFL4/6–ERf complexes reside in xylem initials (Wang *et al.*, 2019). While *er erl2* hypocotyls were indistinguishable from those of the wild type (Wang *et al.*, 2019) and those of *er*f triple mutants were reduced in diameter (Etchells *et al.*, 2013), *er erl1* hypocotyls exhibited a dramatic enhancement of secondary growth (Ikematsu *et al.*, 2017). In wild-type Arabidopsis, radial growth in this organ proceeds in two distinct phases. During the initial ‘proportional phase’, the rate of xylem and phloem formation is roughly equal. The subsequent ‘xylem expansion’ (Fig. 1F) phase is induced by bolting (Sibout *et al.*, 2008), which generates a shoot-derived gibberellic acid (GA) signal (Ragni *et al.*, 2011). The arrival of GA triggers the release of BREVIPEDICELLUS (BP), ARF6, and ARF8 transcription factors from DELLA-mediated repression, shifting the balance of secondary growth to favour xylem production and differentiation of fibres (Ben-Targem *et al.*, 2021). Interestingly, *er erl1* mutation enhanced lignification and expansion of the xylem area to roughly three times that of the wild type (Ikematsu *et al.*, 2017). This suggests that *ER* and *ERL1* ordinarily suppress precocious xylem fibre differentiation during the proportional phase of secondary growth.

Currently, our understanding of signalling components acting downstream of ER in the vasculature is less complete in comparison with stomata. Stomatal clustering in epidermal tissues is regulated by ERf receptors binding to EPF1, EPF2, or EPFL9, in association with SERK co-receptors and the receptor-like protein, TOO MANY MOUTHS (TMM) (Shpak *et al.*, 2005; Lee *et al*., 2012, 2015; Shpak, 2013). Following ligand perception in the epidermis, a MITOGEN-ACTIVATED PROTEIN KINASE (MAPK) cascade is initiated. This culminates in the phosphorylation and destabilization of the transcription factor, SPEECHLESS (SPCH), which specifies the initiation and proliferation of stomatal cells (Jewaria *et al.*, 2013; Meng *et al.*, 2015). In contrast, signalling via EPFL4/6–ERf does not require TMM (Lin *et al.*, 2017). Genetic loss-of-function and expression analyses have highlighted likely transcription factors downstream of this module involved in the regulation of pathogen responses (Cai *et al.*, 2021), inflorescence architecture (Uchida *et al.*, 2012; Meng *et al.*, 2013; Cai *et al.*, 2017), and ovule development (Pillitteri *et al.*, 2007). In hypocotyls, genes encoding the transcriptional regulators of xylem differentiation, *NAC SECONDARY WALL THICKENING PROMOTING FACTOR 1* (*NST1*) and *NST3*, were up-regulated in *er erl1* mutants (Ikematsu *et al.*, 2017), making these likely downstream targets of ER/ERL1 signalling in the xylem. Beyond this suppression of NAC transcription factors, the molecular signalling events underlying ER-mediated inhibition of secondary growth remain elusive. Further targets acting downstream of EPFL4/6–ERf complexes may help explain, for example, the contrasting vascular phenotypes of *er*f and *er erl1* hypocotyls.

## The PXY and ERECTA families genetically interact

In addition to their individual characterization, understanding the crosstalk between RLK-mediated signalling pathways is an important focus of ongoing vascular development research (Fukuda and Hardtke, 2020). ER was recently found to physically interact with another LRR RLK, SUPPRESSOR OF BIR-1 (SOBIR1), and, together, these two receptors signal to suppress precocious fibre development in hypocotyls (Milhinhos *et al.*, 2019). The EPFL4/6–ERf module is also known to genetically interact with TDIF–PXf to control cell proliferation, cell size, and organization within the plant vasculature (Etchells *et al.*, 2013; Uchida and Tasaka, 2013; Wang *et al.*, 2019). When the functions of PXY receptors were removed in *pxy* single or *px*f triple mutants, hypocotyl vascular tissue developed in a disorganized manner, as exemplified by the occurrence of non-periclinal cell divisions (Etchells *et al.*, 2013; Wang *et al.*, 2019). In these same studies, the vasculature of *er* or *er*f hypocotyls exhibited wild-type organization. Interestingly, when both PXf and ERf function were simultaneously compromised, disruption to vascular organization became more pronounced and mean hypocotyl radius was reduced beyond that of *px*f, to the extent that secondary growth initiation was absent in *px*f *er*f sextuple mutants (Etchells *et al.*, 2013; Wang *et al.*, 2019). Similarly, *px*f *er* stem vascular bundles carried fewer cells than the wild type and displayed a dramatically altered shape owing to reduced expansion along the radial axis—a phenotype that was absent in *pxy* and *er* mutants (Uchida and Tasaka, 2013; Wang *et al.*, 2019). Given that the introduction of *er*f mutation(s) in a *px*f background resulted in non-additive enhancement of organizational defects, this is indicative of a synergistic interaction between the two receptor families.

The PXf–ERf genetic interaction could be underpinned by multiple non-mutually exclusive phenomena at the molecular level. Firstly, PXf signalling may regulate the expression of ERf signalling components and vice versa, which could result in compensatory expression when receptors from one family are removed. In corroboration of this hypothesis, the expression of *ERL1* and *ERL2* was up-regulated in *px*f *er* hypocotyls, suggesting that PXf and ER may ordinally interact to suppress *ERL* gene expression (Wang *et al.*, 2019). In surprising contrast, evidence suggests that PXf and ER jointly promote *EPFL6*, *EPFL4*, *ERL1*, and *ERL2* expression in inflorescence stems, highlighting the organ-specific nature of this interaction (Wang *et al.*, 2019). Secondly, PXf and ERf receptors may form protein complexes at the plasma membrane, and the activity of these complexes may be unequally perturbed when different receptor combinations are removed. The expression patterns of *ER* and *PXY* consistently overlap on the xylem side of the cambium in hypocotyls (Hirakawa *et al.*, 2008; Ikematsu *et al.*, 2017; Wang *et al.*, 2019). Furthermore, a recent high-throughput screen for *in vitro* interactions between RLK LRR domains supported the binding of ER to PXY and PXL1, as well as binding of PXL2 to ERL2 (Smakowska-Luzan *et al.*, 2018; Mott *et al.*, 2019).

Thirdly, it is possible that PXf and ERf interact via convergence of their signalling pathways on common genes or proteins. In this scenario, PXf- or ERf-mediated regulation of these hypothetical targets persists in the absence of one receptor family, yet removal of both families abolishes this regulation and gives rise to an enhanced phenotype. With the identification of SERK co-receptors (Zhang *et al.*, 2016*b*) and GSK3 proteins (Kondo *et al.*, 2014; Han *et al.*, 2018), the molecular signalling components transducing PXY signalling to the nucleus are partially understood. Additionally, the NAC transcription factor XYLEM DIFFERENTIATION, DISRUPTION OF VASCULAR PATTERNING (XVP) was recently shown to negatively regulate TDIF–PXY outputs by binding BRI1-ASSOCIATED RECEPTOR KINASE1 (BAK1/SERK3) at the plasma membrane and thus the BAK1–PXY heterodimer (Yang *et al.*, 2020*a*). In contrast, components acting downstream of ER to regulate vascular proliferation are unknown. It is therefore possible that PXY and ER could have shared targets within their signalling cascades, and/or at the level of transcription. The low number of genes known to play a role in the regulation of cambial morphogenesis and maintenance suggests that further regulatory components await discovery (Nieminen *et al.*, 2015; Lehmann and Hardtke, 2016). Indeed, only a few genetic repressors of vascular development have been identified to date (Gursanscky *et al.*, 2016; Ikematsu *et al.*, 2017; Zhang *et al.*, 2019; Wallner *et al.*, 2020; Yang *et al.*, 2020*a*). Given that interactions between the PXf, Erf, and phytohormones evidently make a significant contribution to cambial regulation (Qiang *et al.*, 2013; Wang *et al.*, 2019; Smit *et al.*, 2020), identification of the molecular mechanisms linking these components will be an interesting focus for future research.

## Signalling crosstalk in cambium development requires further study

Crosstalk and interactions between developmental regulators has emerged as a feature of cambium development (Fig. 2C). *LBD4*, described above, is a prime example of this, being regulated by both cytokinin and PXY signalling, as is the genetic interaction between PXf and ERf. Further examples include ethylene signalling converging on *WOX4* (generally considered to act downstream of PXY) to regulate cambial proliferation, while simultaneously inhibiting xylem fibre development through suppression of NAC transcription factors (Yang *et al.*, 2020*b*). In stems, overexpression of another PXY target, *WOX14*, promoted accumulation of bioactive GA, inducing strong lignification of secondary xylem (Denis *et al.*, 2017). Crosstalk between TDIF–PXY and auxin is prevalent in the cambium, as auxin-induced promotion of cell division was reduced in *wox4* stems (Suer *et al.*, 2011). Expression analysis suggested that *PXY* and *WOX4* were transcriptionally regulated by both auxin-responsive MP and HD-ZIP III transcription factors in the root procambium (Smetana *et al.*, 2019). However, MP activity in stems ordinarily inhibits cytokinin biosynthesis and vascular proliferation via activation of *ARABIDOPSIS RESPONSE REGULATOR 7* (*ARR7*) and *ARR15* expression (Han *et al.*, 2018), and suppression of *WOX4* (Brackmann *et al.*, 2018). PXY signalling is thought to remove this inhibition by repressing the kinase activity of the GSK3, BIN2-LIKE 1 (BIL1), a positive regulator of MP function (Han *et al.*, 2018). Overall, it is evident that the coordinated action of multiple factors is crucial for regulating the rate and organization of secondary growth. In future, additional phytohormones such as strigolactone and jasmonic acid, which have been independently identified as positive regulators of secondary growth (Sehr *et al.*, 2010; Agusti *et al.*, 2011*a*), will probably be drawn in to complete the picture.

## Mechanisms of cambial regulation are conserved

As previously discussed, some factors (e.g. MP and ERf) reportedly have organ-specific roles in the Arabidopsis stem and root. Nevertheless, key signalling components, such as PXY–TDIF and cytokinin, function similarly across the vascular system and may therefore represent conserved organizers and drivers of secondary growth. Indeed, there is increasing evidence that these components also regulate wood formation in divergent plant lineages. Tree species in the genus *Populus* and their hybrids (encompassing poplars, aspens, and cottonwoods) have been employed as models for understanding wood formation owing to their relatively rapid growth and availability of genomic resources (Tuskan *et al.*, 2006). Through *in vitro* propagation and transformation of *Populus* spp. (for examples, see Takata and Eriksson, 2012; Maheshwari and Kovalchuk, 2016; Li *et al.*, 2017), combined with tissue-specific transcriptomics and hormone profiling (Immanen *et al.*, 2016; Sundell *et al.*, 2017), researchers have dissected the roles of vascular development regulators in trees.

The three major phytohormone classes implicated in Arabidopsis secondary growth (auxin, cytokinin, and GAs), are also implicated in wood formation. For instance, GA signalling is crucial for triggering the xylem expansion phase of secondary growth of Arabidopsis hypocotyls (Ragni *et al.*, 2011; Ben-Targem *et al.*, 2021). Bioactive GA_4_ peaks in the developing xylem of *Populus trichocarpa* (Immanen *et al.*, 2016), and expression of *Pinus densiflora* GA20-oxidase (*PdGA20ox1*) under constitutive or xylem-specific promoters triggered increased xylem width and cell number in hybrid poplar (Jeon *et al.*, 2016). Together, this highlights a conserved role for GA in regulating secondary xylem expansion.

Reminiscent of models in the Arabidopsis root, auxin and HD-ZIP III transcription factors drive cambial proliferation in *Populus*. In cryosections of hybrid aspen stems (*Populus tremula×tremuloides*), a gradient of IAA (a major bioactive auxin) was detected in developing vascular tissue, with a peak in the cambium and decreasing concentration on either side (Tuominen *et al.*, 1997). Disruption of auxin signalling by ectopic expression of IAA biosynthesis genes or constitutive reduction of auxin responsiveness led to reduced cambial cell division (Tuominen *et al.*, 1997; Nilsson *et al.*, 2008). In addition, tissue-specific transcriptomic analysis identified auxin-responsive genes whose expression patterns correlated with the phytohormone gradient (Nilsson *et al.*, 2008; Immanen *et al.*, 2016). Among these was *PttHB8*, an orthologue of the Arabidopsis HD-ZIP III transcription factor gene, *ATHB8*. Interestingly, *popREVOLUTA* (PRE), the *Populus* orthologue of Arabidopsis *REV*, was up-regulated following the transition of stems to secondary growth, while expression of an miRNA-resistant form of *PRE* resulted in aberrant vascular patterning, including polarity defects and ectopic cambium initiation (Robischon *et al.*, 2011). This is consistent with the role of the HD-ZIP III class in cambial stem cell organization (Smetana *et al.*, 2019). Auxin gradients with peak concentrations in the vascular cambium were similarly detected in *Pinus sylvestris* (Uggla *et al.*, 1996), suggesting that auxin may be important for defining the zone of cambial cell division in both gymnosperms and angiosperms.

Unsurprisingly, auxin’s role in wood formation is interlinked with that of cytokinin. Hormonal profiling and RNA-sequencing of *Populus* stems revealed a peak in cytokinin concentration, biosynthesis, and signalling in the developing phloem (Immanen *et al.*, 2016; Fu *et al.*, 2021). Transgenic hybrid aspen overexpressing the Arabidopsis cytokinin biosynthesis gene, *AtIPT7*, in the cambium and developing xylem contained more cambial stem cells than the wild type and displayed an increased cambial auxin concentration (Immanen *et al.*, 2016). Conversely, cytokinin catabolism was achieved in *Populus* vascular tissue by localizing expression of Arabidopsis *CYTOKININ OXIDASE 2* (*AtCKX2*) to either the cambium or the phloem (Nieminen *et al.*, 2008; Fu *et al.*, 2021). In both cases, this resulted in a reduction in cambial cell division. Phloem- and cambium-localized cytokinin signalling are therefore thought to drive wood formation synergistically.

While cytokinin is a conserved positive regulator of secondary growth, the observation that cytokinin signalling peaks in the tree phloem does not align fully with Arabidopsis root models, in which the cytokinin response is greatest in the procambium (Bishopp *et al.*, 2011). Indeed, the regulation of cytokinin signalling is hypothesized to be more complex in *Populus* spp. owing to the expansion of gene families associated with negative regulation of cytokinin responses (Tuskan *et al.*, 2006). Downstream of cytokinin in Arabidopsis, *LBD1*, *LBD3*, *LBD4*, and *LBD11* drive cambial proliferation in roots (Ye *et al.*, 2021). *LBD* genes were independently identified as positive regulators of wood formation in trees, as *P. tremula×alba* trees overexpressing *PtaLBD1* had thicker stems and enhanced secondary phloem production compared with the wild type (Yordanov *et al.*, 2010). In fact, four *PtaLBD* family members are highly expressed in *Populus* stems undergoing secondary growth, and *PtaLBD1* expression specifically localized to the phloem and adjacent region of the cambial zone (Yordanov *et al.*, 2010), a pattern resembling that of *AtLBD4* (Fig. 3C). Whether or not *PtaLBD* genes are cytokinin responsive or mediate cell growth like their Arabidopsis orthologues remains to be determined.

Alongside those of phytohormones, the roles of TDIF–PXY in promoting and organizing secondary growth are conserved between Arabidopsis and *Populus* spp. Within the *P. trichocarpa* genome, six genes encode putative TDIF-like peptides and four of these (encoded by *PtCLE41a–d*) are specifically expressed in secondary phloem (Kucukoglu *et al.*, 2017). Genes encoding the LRR RLKs, *PtPXYa* and *PtPXYb*, and homeodomain transcription factors, *PtWOX4a* and *PtWOX4b*, were also identified as orthologues of *AtPXY* and *AtWOX4*, respectively. Promoter activity of these four genes peaked in the vascular cambium and positively correlated with cambial activity throughout cycles of growth and dormancy (Kucukoglu *et al.*, 2017). Furthermore, overexpression of *PttCLE41b* and *PttPXYa* in the phloem and cambium, respectively, resulted in a dramatic increase in xylem cell numbers, stem thickening, and biomass formation in hybrid aspen (Etchells *et al.*, 2015). In contrast, hybrid aspen expressing an RNAi construct targeting *PttWOXa/b* suffered severely compromised radial growth, to the extent that they failed to support their own weight (Kucukoglu *et al.*, 2017). Combined with observations that *35S::PttPXYa* constructs complemented Arabidopsis *pxy* mutants (Etchells *et al.*, 2015), this suggests that TDIF–PXY signalling functions similarly in both Arabidopsis and trees to maintain the vascular cambium and promote secondary growth.

Beyond *Populus*, orthologues with similar spatial expression patterns to *AtCLE41*, *AtPXY*, and *AtWOX4* were identified in the gymnosperm tree, *Pinus abies* (Kucukoglu *et al.*, 2017). Additionally, tissue-specific transcriptomics in radish (*Raphanus sativus*), followed by *in situ* hybridization, revealed restricted expression of *RsHB8*, *RsWOX4a*, and *RsPXY* to a subset of root cambial cell layers (Hoang *et al.*, 2020*a*). Further orthologues of established vascular development regulators, including *RsMP*, *RsTMO5*, *RsLHWs*, *RsDOF2.1*, and *RsTMO6*, showed cambium-enriched expression. It is thus possible that both TDIF–PXY and auxin-responsive regulatory pathways coordinate radial growth in herbaceous weeds, trees, and domesticated root crops.

## Perspectives

In the last few decades, our understanding of plant radial growth has been greatly enhanced by pioneering studies that have revealed a complex web of interacting phytohormones and peptide signalling modules. However, several outstanding questions remain. What are the downstream targets of ERf signalling in the vasculature? What is the molecular basis of the PXf–ERf genetic interaction? How is TDIF processed and exported from phloem cells? How is vascular development regulated during times of stress? Interestingly, within their gene regulatory network of Arabidopsis and radish cambium-enriched transcription factors, Hoang *et al.* (2020*a*) noticed over-representation of several stress-responsive genes, including those in the *WRKY* and *ETHYLENE RESPONSE FACTOR* (*ERF*) family. As ethylene positively regulates secondary growth (Etchells *et al.*, 2012; Yang *et al.*, 2020*b*) and acts as an abiotic and biotic stress signal (Dubois *et al.*, 2018; Sasidharan *et al.*, 2018; Riyazuddin *et al.*, 2020), the ethylene response represents a potential pathway whereby secondary growth regulation and stress signalling could be integrated. Future exploration of these open questions may lead to the identification of novel regulatory components and promising intervention points for targeted enhancement of plant secondary growth.

There is increasing evidence that studies of Arabidopsis secondary growth are informative when seeking to understand and manipulate wood formation in trees. However, gene family expansion has been a major contributor to *Populus* biology, and these plants therefore carry considerably more protein-encoding genes than Arabidopsis (Tuskan *et al.*, 2006). For instance, there are 57 *LBD* family genes in *P. trichocarpa* (Zhu *et al.*, 2007), while only 43 are present in Arabidopsis (Shuai *et al.*, 2002). An example of genetic neofunctionalization following duplication in the *Populus* genome could be that of *PtCLE47*, which is a close relative of *AtCLE25*. In Arabidopsis, CLE25 peptides are expressed in phloem cell lineages and signal via CLAVATA 2 (CLV2) receptors to promote phloem initiation (Ren *et al.*, 2019). Surprisingly, *PtCLE47* is expressed in the cambium and drives the formation of secondary xylem (Kucukoglu *et al.*, 2020). It is thus important to consider that both increased genetic redundancy and the evolution of novel regulators may underpin secondary growth in trees.

Collectively, plants contribute a huge proportion (~80%) of the world’s biomass, with stems and tree trunks alone thought to contribute 70% of this (Bar-On *et al.*, 2018). The secondary vascular tissues of trees therefore represents a significant carbon sink and must play a major part in efforts to slow global warming (Bastin *et al.*, 2019). Radial growth also underlies the swelling of root and tuber crops, which provide vital carbohydrates in human diets worldwide (Chandrasekara and Kumar, 2016). While this review has focused on a somewhat esoteric weed, studies in trees and root vegetables demonstrate that discoveries made in Arabidopsis may nevertheless contribute to the enhancement of forestry and agricultural outputs.

## Funding

JPE gratefully acknowledges support from the Biotechnology and Biological Sciences Research Council (Grant: BB/V008129/1).

## References

[CIT0001] Abrash EB , Davies KA, Bergmann DC. 2011. Generation of signaling specificity in Arabidopsis by spatially restricted buffering of ligand–receptor interactions. The Plant cell 23, 2864–2879. 21862708 10.1105/tpc.111.086637 PMC3180797

[CIT0002] Agusti J , Herold S, Schwarz M, et al. 2011a. Strigolactone signaling is required for auxin-dependent stimulation of secondary growth in plants. Proceedings of the National Academy of Sciences, USA 108, 20242–20247. 10.1073/pnas.1111902108 PMC3250165 22123958

[CIT0003] Agusti J , Lichtenberger R, Schwarz M, Nehlin L, Greb T. 2011b. Characterization of transcriptome remodeling during cambium formation identifies MOL1 and RUL1 as opposing regulators of secondary growth. PLoS Genetics 7, e1001312. 21379334 10.1371/journal.pgen.1001312 PMC3040665

[CIT0004] Bagdassarian KS , Brown CM, Jones ET, Etchells P. 2020. Connections in the cambium, receptors in the ring. Current Opinion in Plant Biology 57, 96–103. 32866742 10.1016/j.pbi.2020.07.001

[CIT0005] Bar-On YM , Phillips R, Milo R. 2018. The biomass distribution on Earth. Proceedings of the National Academy of Sciences, USA 115, 6506–6511. 10.1073/pnas.1711842115 PMC6016768 29784790

[CIT0006] Bastin JF , Finegold Y, Garcia C, Mollicone D, Rezende M, Routh D, Zohner CM, Crowther TW. 2019. The global tree restoration potential. Science 365, 76–79. 31273120 10.1126/science.aax0848

[CIT0007] Baum SF , Dubrovsky JG, Rost TL. 2002. Apical organization and maturation of the cortex and vascular cylinder in *Arabidopsis thaliana* (Brassicaceae) roots. American Journal of Botany 89, 908–920. 21665690 10.3732/ajb.89.6.908

[CIT0008] Becraft PW. 2002. Receptor kinase signaling in plant development. Annual Review of Cell and Developmental Biology 18, 163–192. 10.1146/annurev.cellbio.18.012502.083431 12142267

[CIT0009] Ben-Targem M , Ripper D, Bayer M, Ragni L. 2021. Auxin and gibberellin signaling cross-talk promotes hypocotyl xylem expansion and cambium homeostasis. Journal of Experimental Botany 72, 3647–3660. 33619529 10.1093/jxb/erab089

[CIT0010] Bishopp A , Help H, El-Showk S, Weijers D, Scheres B, Friml J, Benková E, Mähönen AP, Helariutta Y. 2011. A mutually inhibitory interaction between auxin and cytokinin specifies vascular pattern in roots. Current Biology 21, 917–926. 21620702 10.1016/j.cub.2011.04.017

[CIT0011] Bollhöner B , Prestele J, Tuominen H. 2012. Xylem cell death: emerging understanding of regulation and function. Journal of Experimental Botany 63, 1081–1094. 22213814 10.1093/jxb/err438

[CIT0012] Bossinger G , Spokevicius AV. 2018. Sector analysis reveals patterns of cambium differentiation in poplar stems. Journal of Experimental Botany 69, 4339–4348. 29931329 10.1093/jxb/ery230 PMC6093462

[CIT0013] Brackmann K , Qi J, Gebert M, et al. 2018. Spatial specificity of auxin responses coordinates wood formation. Nature Communications 9, 875. 10.1038/s41467-018-03256-2 PMC5830446 29491423

[CIT0014] Busch W , Miotk A, Ariel FD, et al. 2010. Transcriptional control of a plant stem cell niche. Developmental Cell 18, 849–861. 20493817 10.1016/j.devcel.2010.03.012

[CIT0015] Cai H , Huang Y, Chen F, Liu L, Chai M, Zhang M, Yan M, Aslam M, He Q, Qin Y. 2021. ERECTA signaling regulates plant immune responses via chromatin-mediated promotion of WRKY33 binding to target genes. New Phytologist 230, 737–756. 10.1111/nph.17200 33454980

[CIT0016] Cai H , Zhao L, Wang L, Zhang M, Su Z, Cheng Y, Zhao H, Qin Y. 2017. ERECTA signaling controls Arabidopsis inflorescence architecture through chromatin-mediated activation of PRE1 expression. New Phytologist 214, 1579–1596. 10.1111/nph.14521 28295392

[CIT0017] Campilho A , Nieminen K, Ragni L. 2020. The development of the periderm: the final frontier between a plant and its environment. Current Opinion in Plant Biology 53, 10–14. 31593816 10.1016/j.pbi.2019.08.008

[CIT0018] Carlsbecker A , Lee JY, Roberts CJ, et al. 2010. Cell signalling by microRNA165/6 directs gene dose-dependent root cell fate. Nature 465, 316–321. 20410882 10.1038/nature08977 PMC2967782

[CIT0019] Chaffey N , Cholewa E, Regan S, Sundberg B. 2002. Secondary xylem development in Arabidopsis: a model for wood formation. Physiologia Plantarum 114, 594–600. 11975734 10.1034/j.1399-3054.2002.1140413.x

[CIT0020] Chandrasekara A , Josheph Kumar T. 2016. Roots and tuber crops as functional foods: a review on phytochemical constituents and their potential health benefits. International Journal of Food Science 2016, 3631647. 27127779 10.1155/2016/3631647 PMC4834168

[CIT0021] Chen MK , Wilson RL, Palme K, Ditengou FA, Shpak ED. 2013. ERECTA family genes regulate auxin transport in the shoot apical meristem and forming leaf primordia. Plant Physiology 162, 1978–1991. 23821653 10.1104/pp.113.218198 PMC3729776

[CIT0022] De Rybel B , Adibi M, Breda AS, et al. 2014. Plant development. Integration of growth and patterning during vascular tissue formation in Arabidopsis. Science 345, 1255215. 25104393 10.1126/science.1255215

[CIT0023] De Rybel B , Mähönen AP, Helariutta Y, Weijers D. 2016. Plant vascular development: from early specification to differentiation. Nature Reviews. Molecular Cell Biology 17, 30–40. 26580717 10.1038/nrm.2015.6

[CIT0024] De Rybel B , Möller B, Yoshida S, Grabowicz I, Barbier de Reuille P, Boeren S, Smith RS, Borst JW, Weijers D. 2013. A bHLH complex controls embryonic vascular tissue establishment and indeterminate growth in Arabidopsis. Developmental Cell 24, 426–437. 23415953 10.1016/j.devcel.2012.12.013

[CIT0025] Denis E , Kbiri N, Mary V, Claisse G, Conde E Silva N, Kreis M, Deveaux Y. 2017. WOX14 promotes bioactive gibberellin synthesis and vascular cell differentiation in Arabidopsis. The Plant Journal 90, 560–572. 28218997 10.1111/tpj.13513

[CIT0026] Dubois M , Van den Broeck L, Inzé D. 2018. The pivotal role of ethylene in plant growth. Trends in Plant Science 23, 311–323. 29428350 10.1016/j.tplants.2018.01.003 PMC5890734

[CIT0027] Esau K. 1977. Anatomy of seed plants. New York: John Wiley & Sons, Ltd.

[CIT0028] Etchells JP , Mishra LS, Kumar M, Campbell L, Turner SR. 2015. Wood formation in trees is increased by manipulating PXY-regulated cell division. Current Biology 25, 1050–1055. 25866390 10.1016/j.cub.2015.02.023 PMC4406943

[CIT0029] Etchells JP , Provost CM, Mishra L, Turner SR. 2013. WOX4 and WOX14 act downstream of the PXY receptor kinase to regulate plant vascular proliferation independently of any role in vascular organisation. Development 140, 2224–2234. 23578929 10.1242/dev.091314 PMC3912870

[CIT0030] Etchells JP , Provost CM, Turner SR. 2012. Plant vascular cell division is maintained by an interaction between PXY and ethylene signalling. PLoS Genetics 8, e1002997. 23166504 10.1371/journal.pgen.1002997 PMC3499249

[CIT0031] Etchells JP , Smit ME, Gaudinier A, Williams CJ, Brady SM. 2016. A brief history of the TDIF–PXY signalling module: balancing meristem identity and differentiation during vascular development. New Phytologist 209, 474–484. 10.1111/nph.13642 26414535

[CIT0032] Etchells JP , Turner SR. 2010a. The PXY–CLE41 receptor ligand pair defines a multifunctional pathway that controls the rate and orientation of vascular cell division. Development 137, 767–774. 20147378 10.1242/dev.044941

[CIT0033] Etchells JP , Turner SR. 2010b. Orientation of vascular cell divisions in Arabidopsis. Plant Signaling & Behavior 5, 730–732. 20404542 10.4161/psb.5.6.11665 PMC3001573

[CIT0034] Fischer U , Kucukoglu M, Helariutta Y, Bhalerao RP. 2019. The dynamics of cambial stem cell activity. Annual Review of Plant Biology 70, 293–319. 10.1146/annurev-arplant-050718-100402 30822110

[CIT0035] Fisher K , Turner S. 2007. PXY, a receptor-like kinase essential for maintaining polarity during plant vascular-tissue development. Current Biology 17, 1061–1066. 17570668 10.1016/j.cub.2007.05.049

[CIT0036] Fu X , Su H, Liu S, Du X, Xu C, Luo K. 2021. Cytokinin signaling localized in phloem noncell-autonomously regulates cambial activity during secondary growth of Populus stems. New Phytologist 230, 1476–1488. 10.1111/nph.17255 33540480

[CIT0037] Fukuda H , Hardtke CS. 2020. Peptide signaling pathways in vascular differentiation. Plant Physiology 182, 1636–1644. 31796560 10.1104/pp.19.01259 PMC7140915

[CIT0038] Furuta KM , Yadav SR, Lehesranta S, et al. 2014. Plant development. Arabidopsis NAC45/86 direct sieve element morphogenesis culminating in enucleation. Science 345, 933–937. 25081480 10.1126/science.1253736

[CIT0039] Gursanscky NR , Jouannet V, Grünwald K, Sanchez P, Laaber-Schwarz M, Greb T. 2016. MOL1 is required for cambium homeostasis in Arabidopsis. The Plant Journal 86, 210–220. 26991973 10.1111/tpj.13169 PMC5021142

[CIT0040] Han S , Cho H, Noh J, Qi J, Jung HJ, Nam H, Lee S, Hwang D, Greb T, Hwang I. 2018. BIL1-mediated MP phosphorylation integrates PXY and cytokinin signalling in secondary growth. Nature Plants 4, 605–614. 29988154 10.1038/s41477-018-0180-3

[CIT0041] Hardtke CS , Berleth T. 1998. The Arabidopsis gene MONOPTEROS encodes a transcription factor mediating embryo axis formation and vascular development. The EMBO Journal 17, 1405–1411. 9482737 10.1093/emboj/17.5.1405 PMC1170488

[CIT0042] He JX , Gendron JM, Yang Y, Li J, Wang ZY. 2002. The GSK3-like kinase BIN2 phosphorylates and destabilizes BZR1, a positive regulator of the brassinosteroid signaling pathway in Arabidopsis. Proceedings of the National Academy of Sciences, USA 99, 10185–10190. 10.1073/pnas.152342599 PMC126645 12114546

[CIT0043] Hilleary R , Gilroy S. 2018. Systemic signaling in response to wounding and pathogens. Current Opinion in Plant Biology 43, 57–62. 29351871 10.1016/j.pbi.2017.12.009

[CIT0044] Hirakawa Y , Kondo Y, Fukuda H. 2010. TDIF peptide signaling regulates vascular stem cell proliferation via the WOX4 homeobox gene in Arabidopsis. The Plant Cell 22, 2618–2629. 20729381 10.1105/tpc.110.076083 PMC2947162

[CIT0045] Hirakawa Y , Shinohara H, Kondo Y, Inoue A, Nakanomyo I, Ogawa M, Sawa S, Ohashi-Ito K, Matsubayashi Y, Fukuda H. 2008. Non-cell-autonomous control of vascular stem cell fate by a CLE peptide/receptor system. Proceedings of the National Academy of Sciences, USA 105, 15208–15213. 10.1073/pnas.0808444105 PMC2567516 18812507

[CIT0046] Hoang NV , Choe G, Zheng Y, et al. 2020a. Identification of conserved gene-regulatory networks that integrate environmental sensing and growth in the root cambium. Current Biology 30, 2887–2900. 32531282 10.1016/j.cub.2020.05.046

[CIT0047] Hoang NV , Park C, Kamran M, Lee JY. 2020b. Gene regulatory network guided investigations and engineering of storage root development in root crops. Frontiers in Plant Science 11, 762. 32625220 10.3389/fpls.2020.00762 PMC7313660

[CIT0048] Ikematsu S , Tasaka M, Torii KU, Uchida N. 2017. ERECTA-family receptor kinase genes redundantly prevent premature progression of secondary growth in the Arabidopsis hypocotyl. New Phytologist 213, 1697–1709. 10.1111/nph.14335 27891614

[CIT0049] Immanen J , Nieminen K, Smolander OP, et al. 2016. Cytokinin and auxin display distinct but interconnected distribution and signaling profiles to stimulate cambial activity. Current Biology 26, 1990–1997. 27426519 10.1016/j.cub.2016.05.053

[CIT0050] Ito Y , Nakanomyo I, Motose H, Iwamoto K, Sawa S, Dohmae N, Fukuda H. 2006. Dodeca-CLE peptides as suppressors of plant stem cell differentiation. Science 313, 842–845. 16902140 10.1126/science.1128436

[CIT0051] Jeon HW , Cho JS, Park EJ, Han KH, Choi YI, Ko JH. 2016. Developing xylem-preferential expression of PdGA20ox1, a gibberellin 20-oxidase 1 from *Pinus densiflora*, improves woody biomass production in a hybrid poplar. Plant Biotechnology Journal 14, 1161–1170. 26503830 10.1111/pbi.12484 PMC11388887

[CIT0052] Jewaria PK , Hara T, Tanaka H, Kondo T, Betsuyaku S, Sawa S, Sakagami Y, Aimoto S, Kakimoto T. 2013. Differential effects of the peptides Stomagen, EPF1 and EPF2 on activation of MAP kinase MPK6 and the SPCH protein level. Plant & Cell Physiology 54, 1253–1262. 23686240 10.1093/pcp/pct076

[CIT0053] Ji J , Strable J, Shimizu R, Koenig D, Sinha N, Scanlon MJ. 2010. WOX4 promotes procambial development. Plant Physiology 152, 1346–1356. 20044450 10.1104/pp.109.149641 PMC2832261

[CIT0054] Johns S , Hagihara T, Toyota M, Gilroy S. 2021. The fast and the furious: rapid long-range signaling in plants. Plant Physiology 185, 694–706. 33793939 10.1093/plphys/kiaa098 PMC8133610

[CIT0055] Jordá L , Sopeña-Torres S, Escudero V, Nuñez-Corcuera B, Delgado-Cerezo M, Torii KU, Molina A. 2016. ERECTA and BAK1 receptor like kinases interact to regulate immune responses in Arabidopsis. Frontiers in Plant Science 7, 1–15. 27446127 10.3389/fpls.2016.00897 PMC4923796

[CIT0056] Koizumi K , Hayashi T, Gallagher KL. 2012. SCARECROW reinforces SHORT-ROOT signaling and inhibits periclinal cell divisions in the ground tissue by maintaining SHR at high levels in the endodermis. Plant Signaling & Behavior 7, 1573–1577. 23072993 10.4161/psb.22437 PMC3578895

[CIT0057] Kondo T , Kajita R, Miyazaki A, et al. 2010. Stomatal density is controlled by a mesophyll-derived signaling molecule. Plant & Cell Physiology 51, 1–8. 20007289 10.1093/pcp/pcp180

[CIT0058] Kondo Y , Fujita T, Sugiyama M, Fukuda H. 2015. A novel system for xylem cell differentiation in *Arabidopsis thaliana*. Molecular Plant 8, 612–621. 25624147 10.1016/j.molp.2014.10.008

[CIT0059] Kondo Y , Ito T, Nakagami H, Hirakawa Y, Saito M, Tamaki T, Shirasu K, Fukuda H. 2014. Plant GSK3 proteins regulate xylem cell differentiation downstream of TDIF–TDR signalling. Nature Communications 5, 3504. 10.1038/ncomms4504 24662460

[CIT0060] Kucukoglu M , Chaabouni S, Zheng B, Mähönen AP, Helariutta Y, Nilsson O. 2020. Peptide encoding Populus CLV3/ESR-RELATED 47 (PttCLE47) promotes cambial development and secondary xylem formation in hybrid aspen. New Phytologist 226, 75–85. 10.1111/nph.16331 PMC7065007 31749215

[CIT0061] Kucukoglu M , Nilsson J, Zheng B, Chaabouni S, Nilsson O. 2017. WUSCHEL-RELATED HOMEOBOX4 (WOX4)-like genes regulate cambial cell division activity and secondary growth in Populus trees. New Phytologist 215, 642–657. 10.1111/nph.14631 28609015

[CIT0062] Lee JS , Hnilova M, Maes M, Lin YC, Putarjunan A, Han SK, Avila J, Torii KU. 2015. Competitive binding of antagonistic peptides fine-tunes stomatal patterning. Nature 522, 439–443. 26083750 10.1038/nature14561 PMC4532310

[CIT0063] Lee JS , Kuroha T, Hnilova M, Khatayevich D, Kanaoka MM, McAbee JM, Sarikaya M, Tamerler C, Torii KU. 2012. Direct interaction of ligand–receptor pairs specifying stomatal patterning. Genes & Development 26, 126–136. 22241782 10.1101/gad.179895.111 PMC3273837

[CIT0064] Lee JS , Torii KU. 2012. A tale of two systems: peptide ligand–receptor pairs in plant development. Cold Spring Harbor Symposia on Quantitative Biology 77, 83–89. 23274454 10.1101/sqb.2012.77.014886

[CIT0065] Lehmann F , Hardtke CS. 2016. Secondary growth of the Arabidopsis hypocotyl–vascular development in dimensions. Current Opinion in Plant Biology 29, 9–15. 26667498 10.1016/j.pbi.2015.10.011

[CIT0066] Leyser O. 2018. Auxin signaling. Plant Physiology 176, 465–479. 28818861 10.1104/pp.17.00765 PMC5761761

[CIT0067] Li S , Zhen C, Xu W, Wang C, Cheng Y. 2017. Simple, rapid and efficient transformation of genotype Nisqually-1: a basic tool for the first sequenced model tree. Scientific Reports 7, 2638. 28572673 10.1038/s41598-017-02651-x PMC5453977

[CIT0068] Lin G , Zhang L, Han Z, Yang X, Liu W, Li E, Chang J, Qi Y, Shpak ED, Chai J. 2017. A receptor-like protein acts as a specificity switch for the regulation of stomatal development. Genes & Development 31, 927–938. 28536146 10.1101/gad.297580.117 PMC5458759

[CIT0069] Llorente F , Alonso-Blanco C, Sánchez-Rodriguez C, Jorda L, Molina A. 2005. ERECTA receptor-like kinase and heterotrimeric G protein from Arabidopsis are required for resistance to the necrotrophic fungus *Plectosphaerella cucumerina*. The Plant Journal 43, 165–180. 15998304 10.1111/j.1365-313X.2005.02440.x

[CIT0070] Long Y , Smet W, Cruz-Ramírez A, et al. 2015. Arabidopsis BIRD zinc finger proteins jointly stabilize tissue boundaries by confining the cell fate regulator SHORT-ROOT and contributing to fate specification. The Plant Cell 27, 1185–1199. 25829440 10.1105/tpc.114.132407 PMC4558684

[CIT0071] Long Y , Stahl Y, Weidtkamp-Peters S, et al. 2017. In vivo FRET-FLIM reveals cell-type-specific protein interactions in Arabidopsis roots. Nature 548, 97–102. 28746306 10.1038/nature23317

[CIT0072] Maheshwari P , Kovalchuk I. 2016. Agrobacterium-mediated stable genetic transformation of *Populus angustifolia* and *Populus balsamifera*. Frontiers in Plant Science 7, 296. 27014319 10.3389/fpls.2016.00296 PMC4783574

[CIT0073] Mallory AC , Reinhart BJ, Jones-Rhoades MW, Tang G, Zamore PD, Barton MK, Bartel DP. 2004. MicroRNA control of PHABULOSA in leaf development: importance of pairing to the microRNA 5ʹ region. The EMBO Journal 23, 3356–3364. 15282547 10.1038/sj.emboj.7600340 PMC514513

[CIT0074] Matsumoto-Kitano M , Kusumoto T, Tarkowski P, Kinoshita-Tsujimura K, Václavíková K, Miyawaki K, Kakimoto T. 2008. Cytokinins are central regulators of cambial activity. Proceedings of the National Academy of Sciences, USA 105, 20027–20031. 10.1073/pnas.0805619105 PMC2605004 19074290

[CIT0075] Meng X , Chen X, Mang H, Liu C, Yu X, Gao X, Torii KU, He P, Shan L. 2015. Differential function of Arabidopsis SERK family receptor-like kinases in stomatal patterning. Current Biology 25, 2361–2372. 26320950 10.1016/j.cub.2015.07.068 PMC4714584

[CIT0076] Meng X , Wang H, He Y, Liu Y, Walker JC, Torii KU, Zhang S. 2013. A MAPK cascade downstream of ERECTA receptor-like protein kinase regulates Arabidopsis inflorescence architecture by promoting localized cell proliferation. The Plant Cell 24, 4948–4960. 10.1105/tpc.112.104695 PMC3556968 23263767

[CIT0077] Milhinhos A , Vera-Sirera F, Blanco-Touriñán N, et al. 2019. SOBIR1/EVR prevents precocious initiation of fiber differentiation during wood development through a mechanism involving BP and ERECTA. Proceedings of the National Academy of Sciences, USA 116, 18710–18716. 10.1073/pnas.1807863116 PMC6744917 31444299

[CIT0078] Miyashima S , Roszak P, Sevilem I, et al. 2019. Mobile PEAR transcription factors integrate positional cues to prime cambial growth. Nature 565, 490–494. 30626969 10.1038/s41586-018-0839-y PMC7617008

[CIT0079] Miyashima S , Sebastian J, Lee JY, Helariutta Y. 2013. Stem cell function during plant vascular development. The EMBO Journal 32, 178–193. 23169537 10.1038/emboj.2012.301 PMC3553377

[CIT0080] Miyawaki K , Tarkowski P, Matsumoto-Kitano M, Kato T, Sato S, Tarkowska D, Tabata S, Sandberg G, Kakimoto T. 2006. Roles of Arabidopsis ATP/ADP isopentenyltransferases and tRNA isopentenyltransferases in cytokinin biosynthesis. Proceedings of the National Academy of Sciences, USA 103, 16598–16603. 10.1073/pnas.0603522103 PMC1637627 17062755

[CIT0081] Morita J , Kato K, Nakane T, Kondo Y, Fukuda H, Nishimasu H, Ishitani R, Nureki O. 2016. Crystal structure of the plant receptor-like kinase TDR in complex with the TDIF peptide. Nature Communications 7, 12383. 10.1038/ncomms12383 PMC4979064 27498761

[CIT0082] Mott GA , Smakowska-Luzan E, Pasha A, et al. 2019. Map of physical interactions between extracellular domains of Arabidopsis leucine-rich repeat receptor kinases. Scientific Data 6, 190025. 30806640 10.1038/sdata.2019.25 PMC6390701

[CIT0083] Nakajima K , Sena G, Nawy T, Benfey PN. 2001. Intercellular movement of the putative transcription factor SHR in root patterning. Nature 413, 307–311. 11565032 10.1038/35095061

[CIT0084] Nieminen K , Blomster T, Helariutta Y, Mähönen AP. 2015. Vascular cambium development. The Arabidopsis Book 13, e0177. 26078728 10.1199/tab.0177 PMC4463761

[CIT0085] Nieminen K , Immanen J, Laxell M, et al. 2008. Cytokinin signaling regulates cambial development in poplar. Proceedings of the National Academy of Sciences, USA 105, 20032–20037. 10.1073/pnas.0805617106 PMC2604918 19064928

[CIT0086] Nilsson J , Karlberg A, Antti H, Lopez-Vernaza M, Mellerowicz E, Perrot-Rechenmann C, Sandberg G, Bhalerao RP. 2008. Dissecting the molecular basis of the regulation of wood formation by auxin in hybrid aspen. The Plant Cell 20, 843–855. 18424614 10.1105/tpc.107.055798 PMC2390731

[CIT0087] Ohashi-Ito K , Bergmann DC. 2007. Regulation of the Arabidopsis root vascular initial population by LONESOME HIGHWAY. Development 134, 2959–2968. 17626058 10.1242/dev.006296 PMC3145339

[CIT0088] Ohashi-Ito K , Saegusa M, Iwamoto K, Oda Y, Katayama H, Kojima M, Sakakibara H, Fukuda H. 2014. A bHLH complex activates vascular cell division via cytokinin action in root apical meristem. Current Biology 24, 2053–2058. 25131670 10.1016/j.cub.2014.07.050

[CIT0089] Pillitteri LJ , Bemis SM, Shpak ED, Torii KU. 2007. Haploinsufficiency after successive loss of signaling reveals a role for ERECTA-family genes in Arabidopsis ovule development. Development 134, 3099–3109. 17652352 10.1242/dev.004788

[CIT0090] Qiang Y , Wu J, Han H, Wang G. 2013. CLE peptides in vascular development. Journal of Integrative Plant Biology 55, 389–394. 23473393 10.1111/jipb.12044

[CIT0091] Ragni L , Nieminen K, Pacheco-Villalobos D, Sibout R, Schwechheimer C, Hardtke CS. 2011. Mobile gibberellin directly stimulates Arabidopsis hypocotyl xylem expansion. The Plant cell 23, 1322–1336. 21498678 10.1105/tpc.111.084020 PMC3101547

[CIT0092] Ren SC , Song XF, Chen WQ, Lu R, Lucas WJ, Liu CM. 2019. CLE25 peptide regulates phloem initiation in Arabidopsis through a CLERK–CLV2 receptor complex. Journal of Integrative Plant Biology 61, 1043–1061. 31127689 10.1111/jipb.12846

[CIT0093] Riyazuddin R , Verma R, Singh K, Nisha N, Keisham M, Bhati KK, Kim ST, Gupta R. 2020. Ethylene: a master regulator of salinity stress tolerance in plants. Biomolecules 10, 959. 10.3390/biom10060959 PMC7355584 32630474

[CIT0094] Robischon M , Du J, Miura E, Groover A. 2011. The Populus class III HD ZIP, popREVOLUTA, influences cambium initiation and patterning of woody stems. Plant Physiology 155, 1214–1225. 21205615 10.1104/pp.110.167007 PMC3046580

[CIT0095] Rodriguez-Villalon A , Gujas B, Kang YH, Breda AS, Cattaneo P, Depuydt S, Hardtke CS. 2014. Molecular genetic framework for protophloem formation. Proceedings of the National Academy of Sciences, USA 111, 11551–11556. 10.1073/pnas.1407337111 PMC4128119 25049386

[CIT0096] Sablowski R , Carnier Dornelas M. 2014. Interplay between cell growth and cell cycle in plants. Journal of Experimental Botany 65, 2703–2714. 24218325 10.1093/jxb/ert354

[CIT0097] Saito M , Kondo Y, Fukuda H. 2018. BES1 and BZR1 redundantly promote phloem and xylem differentiation. Plant & Cell Physiology 59, 590–600. 29385529 10.1093/pcp/pcy012

[CIT0098] Sasidharan R , Hartman S, Liu Z, Martopawiro S, Sajeev N, van Veen H, Yeung E, Voesenek LACJ. 2018. Signal dynamics and interactions during flooding stress. Plant Physiology 176, 1106–1117. 29097391 10.1104/pp.17.01232 PMC5813540

[CIT0099] Schlereth A , Möller B, Liu W, Kientz M, Flipse J, Rademacher EH, Schmid M, Jürgens G, Weijers D. 2010. MONOPTEROS controls embryonic root initiation by regulating a mobile transcription factor. Nature 464, 913–916. 20220754 10.1038/nature08836

[CIT0100] Schoof H , Lenhard M, Haecker A, Mayer KF, Jürgens G, Laux T. 2000. The stem cell population of Arabidopsis shoot meristems in maintained by a regulatory loop between the CLAVATA and WUSCHEL genes. Cell 100, 635–644. 10761929 10.1016/s0092-8674(00)80700-x

[CIT0101] Sehr EM , Agusti J, Lehner R, Farmer EE, Schwarz M, Greb T. 2010. Analysis of secondary growth in the Arabidopsis shoot reveals a positive role of jasmonate signalling in cambium formation. The Plant Journal 63, 811–822. 20579310 10.1111/j.1365-313X.2010.04283.x PMC2988407

[CIT0102] Shi D , Lebovka I, Loṕez-Salmeroń V, Sanchez P, Greb T. 2019. Bifacial cambium stem cells generate xylem and phloem during radial plant growth. Development 146, 1–8. 10.1242/dev.171355 PMC6340147 30626594

[CIT0103] Shpak ED. 2013. Diverse roles of ERECTA family genes in plant development. Journal of Integrative Plant Biology 55, 1238–1250. 24016315 10.1111/jipb.12108

[CIT0104] Shpak ED , Berthiaume CT, Hill EJ, Torii KU. 2004. Synergistic interaction of three ERECTA-family receptor-like kinases controls Arabidopsis organ growth and flower development by promoting cell proliferation. Development 131, 1491–1501. 14985254 10.1242/dev.01028

[CIT0105] Shpak ED , McAbee JM, Pillitteri LJ, Torii KU. 2005. Stomatal patterning and differentiation by synergistic interactions of receptor kinases. Science 309, 290–293. 16002616 10.1126/science.1109710

[CIT0106] Shuai B , Reynaga-Peña CG, Springer PS. 2002. The lateral organ boundaries gene defines a novel, plant-specific gene family. Plant Physiology 129, 747–761. 12068116 10.1104/pp.010926 PMC161698

[CIT0107] Sibout R , Plantegenet S, Hardtke CS. 2008. Flowering as a condition for xylem expansion in Arabidopsis hypocotyl and root. Current Biology 18, 458–463. 18356049 10.1016/j.cub.2008.02.070

[CIT0108] Smakowska-Luzan E , Mott GA, Parys K, et al. 2018. An extracellular network of Arabidopsis leucine-rich repeat receptor kinases. Nature 553, 342–346. 29320478 10.1038/nature25184 PMC6485605

[CIT0109] Smet W , Sevilem I, de Luis Balaguer MA, et al. 2019. DOF2.1 controls cytokinin-dependent vascular cell proliferation downstream of TMO5/LHW. Current Biology 29, 520–529. 30686737 10.1016/j.cub.2018.12.041 PMC6370950

[CIT0110] Smetana O , Mäkilä R, Lyu M, et al. 2019. High levels of auxin signalling define the stem-cell organizer of the vascular cambium. Nature 565, 485–489. 30626967 10.1038/s41586-018-0837-0

[CIT0111] Smit ME , McGregor SR, Sun H, et al. 2020. A PXY-mediated transcriptional network integrates signaling mechanisms to control vascular development in Arabidopsis. The Plant Cell 32, 319–335. 31806676 10.1105/tpc.19.00562 PMC7008486

[CIT0112] Spicer R , Groover A. 2010. Evolution of development of vascular cambia and secondary growth. New Phytologist 186, 577–592. 10.1111/j.1469-8137.2010.03236.x 20522166

[CIT0113] Stahl Y , Simon R. 2009. Is the Arabidopsis root niche protected by sequestration of the CLE40 signal by its putative receptor ACR4? Plant Signaling & Behavior 4, 634–635. 19820344 10.4161/psb.4.7.8970 PMC2710560

[CIT0114] Suer S , Agusti J, Sanchez P, Schwarz M, Greb T. 2011. WOX4 imparts auxin responsiveness to cambium cells in Arabidopsis. The Plant Cell 23, 3247–3259. 21926336 10.1105/tpc.111.087874 PMC3203433

[CIT0115] Sugano SS , Shimada T, Imai Y, Okawa K, Tamai A, Mori M, Hara-Nishimura I. 2010. Stomagen positively regulates stomatal density in Arabidopsis. Nature 463, 241–244. 20010603 10.1038/nature08682

[CIT0116] Sundell D , Street NR, Kumar M, et al. 2017. AspWood: high-spatial-resolution transcriptome profiles reveal uncharacterized modularity of wood formation in *Populus tremula*. The Plant Cell 29, 1585–1604. 28655750 10.1105/tpc.17.00153 PMC5559752

[CIT0117] Taiz L , Zeiger E. 2002. Plant physiology. Sunderland, MA: Sinauer Associates Inc.

[CIT0118] Takata N , Eriksson ME. 2012. A simple and efficient transient transformation for hybrid aspen (*Populus tremula* × *P. tremuloides*). Plant Methods 8, 30. 22871142 10.1186/1746-4811-8-30 PMC3476444

[CIT0119] Takata N , Yokota K, Ohki S, Mori M, Taniguchi T, Kurita M. 2013. Evolutionary relationship and structural characterization of the EPF/EPFL gene family. PLoS One 8, e65183. 23755192 10.1371/journal.pone.0065183 PMC3670920

[CIT0120] Tameshige T , Okamoto S, Lee JS, Aida M, Tasaka M, Torii KU, Uchida N. 2016. A secreted peptide and its receptors shape the auxin response pattern and leaf margin morphogenesis. Current Biology 26, 2478–2485. 27593376 10.1016/j.cub.2016.07.014

[CIT0121] Thamm A , Sanegre-Sans S, Paisley J, Meader S, Milhinhos A, Contera S, Agusti J. 2019. A simple mathematical model of allometric exponential growth describes the early three-dimensional growth dynamics of secondary xylem in Arabidopsis roots. Royal Society Open Science 6, 190126. 31032061 10.1098/rsos.190126 PMC6458390

[CIT0122] Tonn N , Greb T. 2017. Radial plant growth. Current Biology 27, R878–R882. 28898657 10.1016/j.cub.2017.03.056

[CIT0123] Torii KU , Mitsukawa N, Oosumi T, Matsuura Y, Yokoyama R, Whittier RF, Komeda Y. 1996. The Arabidopsis ERECTA gene encodes a putative receptor protein kinase with extracellular leucine-rich repeats. The Plant Cell 8, 735–746. 8624444 10.1105/tpc.8.4.735 PMC161133

[CIT0124] Trewavas A. 2021. Awareness and integrated information theory identify plant meristems as sites of conscious activity. Protoplasma 258, 673–679. 33745091 10.1007/s00709-021-01633-1 PMC8052216

[CIT0125] Tuominen H , Puech L, Fink S, Sundberg B. 1997. A radial concentration gradient of indole-3-acetic acid is related to secondary xylem development in hybrid aspen. Plant Physiology 115, 577–585. 12223825 10.1104/pp.115.2.577 PMC158517

[CIT0126] Tuskan GA , Difazio S, Jansson S, et al. 2006. The genome of black cottonwood, *Populus trichocarpa* (Torr. & Gray). Science 313, 1596–1604. 16973872 10.1126/science.1128691

[CIT0127] Uchida N , Lee JS, Horst RJ, Lai HH, Kajita R, Kakimoto T, Tasaka M, Torii KU. 2012. Regulation of inflorescence architecture by intertissue layer ligand–receptor communication between endodermis and phloem. Proceedings of the National Academy of Sciences, USA 109, 6337–6342. 10.1073/pnas.1117537109 PMC3341066 22474391

[CIT0128] Uchida N , Tasaka M. 2013. Regulation of plant vascular stem cells by endodermis-derived EPFL-family peptide hormones and phloem-expressed ERECTA-family receptor kinases. Journal of Experimental Botany 64, 5335–5343. 23881395 10.1093/jxb/ert196

[CIT0129] Uggla C , Moritz T, Sandberg G, Sundberg B. 1996. Auxin as a positional signal in pattern formation in plants. Proceedings of the National Academy of Sciences, USA 93, 9282–9286. 10.1073/pnas.93.17.9282 PMC38633 11607701

[CIT0130] Wallner ES , Tonn N, Shi D, Jouannet V, Greb T. 2020. SUPPRESSOR OF MAX2 1-LIKE 5 promotes secondary phloem formation during radial stem growth. The Plant Journal 102, 903–915. 31910293 10.1111/tpj.14670

[CIT0131] Wang H. 2020. Regulation of vascular cambium activity. Plant Science 291, 110322. 31928672 10.1016/j.plantsci.2019.110322

[CIT0132] Wang J , Kucukoglu M, Zhang L, Chen P, Decker D, Nilsson O, Jones B, Sandberg G, Zheng B. 2013. The Arabidopsis LRR-RLK, PXC1, is a regulator of secondary wall formation correlated with the TDIF–PXY/TDR–WOX4 signaling pathway. BMC Plant Biology 13, 94. 23815750 10.1186/1471-2229-13-94 PMC3716795

[CIT0133] Wang N , Bagdassarian KS, Doherty RE, Kroon JT, Connor KA, Wang XY, Wang W, Jermyn IH, Turner SR, Etchells JP. 2019. Organ-specific genetic interactions between paralogues of the PXY and ER receptor kinases enforce radial patterning in Arabidopsis vascular tissue. Development 146, 177105. 10.1242/dev.177105 31043420

[CIT0134] Weijers D , Schlereth A, Ehrismann JS, Schwank G, Kientz M, Jürgens G. 2006. Auxin triggers transient local signaling for cell specification in Arabidopsis embryogenesis. Developmental Cell 10, 265–270. 16459305 10.1016/j.devcel.2005.12.001

[CIT0135] Whitford R , Fernandez A, De Groodt R, Ortega E, Hilson P. 2008. Plant CLE peptides from two distinct functional classes synergistically induce division of vascular cells. Proceedings of the National Academy of Sciences, USA 105, 18625–18630. 10.1073/pnas.0809395105 PMC2587568 19011104

[CIT0136] Woodward C , Bemis SM, Hill EJ, Sawa S, Koshiba T, Torii KU. 2005. Interaction of auxin and ERECTA in elaborating Arabidopsis inflorescence architecture revealed by the activation tagging of a new member of the YUCCA family putative flavin monooxygenases. Plant Physiology 139, 192–203. 16126863 10.1104/pp.105.063495 PMC1203369

[CIT0137] Wunderling A , Ripper D, Barra-Jimenez A, Mahn S, Sajak K, Targem MB, Ragni L. 2018. A molecular framework to study periderm formation in Arabidopsis. New Phytologist 219, 216–229. 10.1111/nph.15128 29611875

[CIT0138] Yang JH , Lee KH, Du Q, Yang S, Yuan B, Qi L, Wang H. 2020a. A membrane-associated NAC domain transcription factor XVP interacts with TDIF co-receptor and regulates vascular meristem activity. New Phytologist 226, 59–74. 10.1111/nph.16289 31660587

[CIT0139] Yang S , Wang S, Li S, Du Q, Qi L, Wang W, Chen J, Wang H. 2020b. Activation of ACS7 in Arabidopsis affects vascular development and demonstrates a link between ethylene synthesis and cambial activity. Journal of Experimental Botany 71, 7160–7170. 32926140 10.1093/jxb/eraa423

[CIT0140] Ye L , Wang X, Lyu M, Siligato R, Eswaran G, Vainio L, Blomster T, Zhang J, Mähönen AP. 2021. Cytokinins initiate secondary growth in the Arabidopsis root through a set of LBD genes. Current Biology 31, 3365–3373. 34129827 10.1016/j.cub.2021.05.036 PMC8360765

[CIT0141] Yordanov YS , Regan S, Busov V. 2010. Members of the LATERAL ORGAN BOUNDARIES DOMAIN transcription factor family are involved in the regulation of secondary growth in Populus. The Plant Cell 22, 3662–3677. 21097711 10.1105/tpc.110.078634 PMC3015109

[CIT0142] Zhang H , Lin X, Han Z, Qu LJ, Chai J. 2016a. Crystal structure of PXY–TDIF complex reveals a conserved recognition mechanism among CLE peptide–receptor pairs. Cell Research 26, 543–555. 27055373 10.1038/cr.2016.45 PMC4856767

[CIT0143] Zhang H , Lin X, Han Z, Wang J, Qu LJ, Chai J. 2016b. SERK family receptor-like kinases function as co-receptors with PXY for plant vascular development. Molecular Plant 9, 1406–1414. 27449136 10.1016/j.molp.2016.07.004

[CIT0144] Zhang J , Eswaran G, Alonso-Serra J, et al. 2019. Transcriptional regulatory framework for vascular cambium development in Arabidopsis roots. Nature Plants 5, 1033–1042. 31595065 10.1038/s41477-019-0522-9 PMC6795544

[CIT0145] Zhu QH , Guo AY, Gao G, Zhong YF, Xu M, Huang M, Luo J. 2007. DPTF: a database of poplar transcription factors. Bioinformatics 23, 1307–1308. 17392330 10.1093/bioinformatics/btm113

